# 1824. Combination therapy of daptomycin/colistin for extreme drug resistant *Acinetobacter baumannii* bacteremia

**DOI:** 10.1093/ofid/ofac492.1454

**Published:** 2022-12-15

**Authors:** Charis Lampada, Maria Kourti, Konstantina Charisi, Elias Iosifidis, Charalambos Zarras, Emmanuel Roilides, Charalambos Antachopoulos

**Affiliations:** 3rd Department of Pediatrics, Aristotle University of Thessaloniki and Hippokration General Hospital, Thessaloniki, Thessaloniki, Greece; 3rd Department of Pediatrics, Aristotle University of Thessaloniki and Hippokration General Hospital, Thessaloniki, Thessaloniki, Greece; 3rd Department of Pediatrics, Aristotle University of Thessaloniki and Hippokration General Hospital, Thessaloniki, Thessaloniki, Greece; 3rd Department of Pediatrics, Aristotle University of Thessaloniki and Hippokration General Hospital, Thessaloniki, Thessaloniki, Greece; Microbiology Department, Hippokration General Hospital, Thessaloniki, Thesprotia, Greece; 3rd, Thessaloniki, Thessaloniki, Greece; 3rd Department of Pediatrics, Aristotle University of Thessaloniki and Hippokration General Hospital, Thessaloniki, Thessaloniki, Greece

## Abstract

**Background:**

There are experimental data demonstrating synergistic *in-vitro* activity of colistin and daptomycin against extremely drug-resistant (XDR) strains of *Acinetobacter baumannii*.Aim of this study is the description of a case-series of patients with confirmed XDR *A. baumannii* bloodstream infection (BSI) receiving an antimicrobial regimen including colistin and daptomycin.

**Methods:**

Prospective study of patients with BSI caused by XDR *A. baumannii* from May 2021 to February 2022 in a tertiary level hospital. Demographic, clinical and laboratory data were recorded and evaluated from all patients. BacT Alert system (BiomerieuxR) was used for blood cultures and bacterial identification and susceptibility testing was performed using VITEK® 2. Colistin susceptibility testing (dilution method) were interpreted in accordance to Clinical and Laboratory Standards Institute (CLSI) criteria.

**Results:**

A total of 16 episodes of XDR *A. baumannii* were recorded in 16 patients (14 males). Median age was 60 (range 38-85) years. Ten patients (56.2%) were hospitalized in medical wards, and 43.8% in surgery wards. One-third of patients had previously been admitted to the Intensive Care Unit, while all patients had an indwelling central vascular catheter. Fourteen of the 16 XDR strains of *A. baumannii* were also resistant to colistin. Also, 2 of the 16 strains had a MIC of 2μg/ml for tigecycline, while in the remaining 14 the MIC was > 2μg/ml. All isolates had meropenem MIC >16μg/ml. All patients received a combination of colistin -daptomycin -meropenem -tigecycline. Clinical and laboratory improvement was noted in 15 of the 16 patients within the 14 days of treatment. Fifteen out of the 16 patients showed a microbiological response: the mean duration between the start of treatment and the first negative blood culture was 12 days.Finally, no patient experienced a marked increase in CPK due to daptomycin use.

**Conclusion:**

The outcome of this case series of patients receiving colistin-daptomycin combination as part of a broader synergistic antimicrobial regimen to treat XDR *A. baumannii* was satisfactory, with no significant adverse reactions. This potential clinical benefit of the colistin/daptomycin combination should be further evaluated in a larger comparative study.

**Disclosures:**

**All Authors**: No reported disclosures.

